# The Noc-Domain Containing C-Terminus of Noc4p Mediates Both Formation of the Noc4p-Nop14p Submodule and Its Incorporation into the SSU Processome

**DOI:** 10.1371/journal.pone.0008370

**Published:** 2009-12-18

**Authors:** Holger Kühn, Thomas Hierlmeier, Juliane Merl, Steffen Jakob, Almass-Houd Aguissa-Touré, Philipp Milkereit, Herbert Tschochner

**Affiliations:** 1 Institut für Biochemie, Genetik und Mikrobiologie, University of Regensburg, Regensburg, Germany; 2 Laboratoire de Biologie Moléculaire Eucaryote, Université de Toulouse UPS, Toulouse, France; Institute of Genetics and Molecular and Cellular Biology, France

## Abstract

Noc1p, Noc3p and Noc4p are eukaryotic proteins which play essential roles in yeast ribosome biogenesis and contain a homologous stretch of about 45 aminoacids (Noc-domain) of unknown function. Yeast Noc4p is a component of the small ribosomal subunit (SSU) processome, can be isolated as a stable Noc4p-Nop14p SSU-processome submodule from yeast cells, and is required for nuclear steps of small ribosomal subunit rRNA maturation. We expressed a series of mutated alleles of NOC4 in yeast cells and analysed whether the corresponding protein variants support vegetative growth, interact with Nop14p, and are incorporated into the SSU-processome. The data reveal that the essential C-terminus of Noc4p which contains 237 aminoacids including the Noc-domain represents a protein-protein interaction module. It is required and sufficient for its association with Nop14p and several nuclear precursors of the small ribosomal subunit. The N-terminal Noc4-part seems to be targeted to pre-ribosomes via the C-terminus of Noc4p and plays there an essential role in SSU-processome function. Replacement of the Noc4p-Noc-domain by its homologues Noc1p-counterpart results in a hybrid Noc4p variant which fails to associate with Nop14p and pre-ribosomes. On the other hand, exchange of 6 amino acids in the Noc1-Noc-domain of this hybrid Noc4p protein is sufficient to restore its essential *in vivo* functions. These data suggest that Noc-domains of Noc1p and Noc4p share a common structural backbone in which diverging amino acids play crucial roles in mediating specific regulated interactions. Our analysis allows us to distinguish between different functions of certain domains within Noc4p and contribute to the understanding of how incorporation of Noc4p into ribosomal precursors is coupled to rRNA processing and maturation of the small ribosomal subunit.

## Introduction

**Figure 1:**
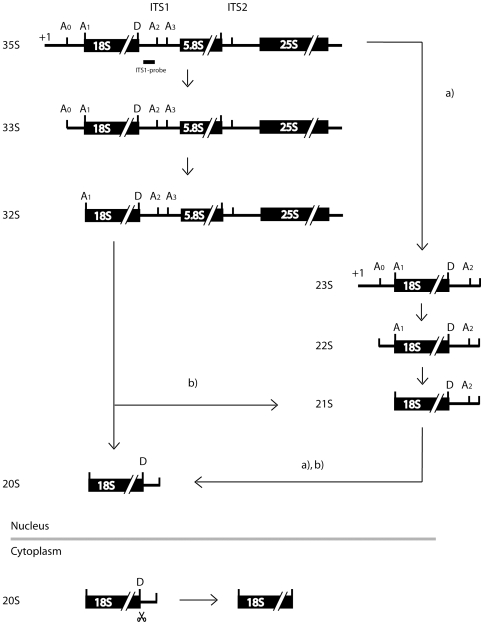
Pathways of 18S rRNA precursor maturation in *S. cerevisiae*. Three of the four rRNA-species found in mature ribosomes are generated from the 35S precursor RNA, which is processed in a series of defined endo- and exonucleolytic cleavages. The scheme shows an overview of 18S rRNA precursors found in *S. cerevisiae* and their suggested interrelationship. A main pre-18S rRNA processing pathway is thought to occur via early consecutive cleavages at sites A0, A1 and A2 leading to 20S pre-rRNA which is cleaved in the cytoplasm at site D to result in 18S rRNA. Alternative pre-18S rRNA processing pathways marked in the figure by letters a) and b) were proposed in [31], [43].

Eukaryotic ribosomes consist of four rRNAs and more than 70 ribosomal proteins (r-proteins). In the yeast *Saccharomyces cerevisiae* the large ribosomal subunit (LSU) contains the 25S, 5.8S and 5S rRNA and 46 r-proteins whereas the small ribosomal subunit (SSU) consists of the 18S rRNA and about 32 r-proteins. Biogenesis of the two eukaryotic ribosomal subunits requires the coordinated action of many proteinaceous factors and small nucleolar RNA (snoRNA) containing ribonucleoprotein particles (RNPs) and proceeds in the nucleolus, nucleoplasm and cytoplasm. Biogenesis factors can be involved in a variety of reactions like rRNA cleavage, other rRNA modifications, RNA folding, assembly of r-proteins, quality control of the nascent ribosome as well as nuclear transport and export to the cytoplasm [1], [2], [3]. It was suggested that the first ribosome biogenesis factors assemble co-transcriptionally as components of the SSU-processome (small subunit processome) resulting in the formation of the terminal knobs on the ends of nascent rRNAs which are visible in electron micrographs of spreaded nucleoli [4], [5]. The SSU-processome is also referred to as the 35S precursor rRNA (pre-rRNA) containing 90S precursor ribosome (pre-ribosome) [6] and consists of the U3 small nucleolar RNA and about 40 U three proteins (UTPs), among them Noc4p, all of which are required for the early cleavage of the pre-rRNA at sites A0, A1 and A2 (see Fig. 1). Large scale proteome-analysis revealed three UTP containing subcomplexes, UTP-A, UTP-B and UTP-C which can be isolated from cellular extracts depleted of pre-ribosomes through differential centrifugation [7]. It was shown, that incorporation of U3 snoRNA, UTP-B and UTP-C into pre-ribosomes requires the functional integrity of UTP-A components [5], [8], [9]. Whether members of the UTP-A subcomplex associate with rDNA chromatin independent on ongoing rRNA synthesis and/or promote efficient rDNA transcription is currently still up for debate [10], [11], [12], [13], [14]. At the time when cleavage of pre-rRNA at A2 occurs, which separates the SSU from the LSU maturation pathway, most of the SSU processome components apparently leave the nascent pre-ribosome [5], [15]. After subsequent formation of increasingly stable r-protein-rRNA assembly intermediates [16] which contain only a few non-ribosomal proteins [15] the pre-40S subunit is rapidly exported through the nuclear pore to the cytoplasm, where the last processing steps including maturation from 20S pre-rRNA to 18S rRNA take place. After cleavage at site A2 a different set of nonribosomal factors binds to the resulting 27SA_2_ pre-rRNA containing RNPs to generate pre-60S particles. Several LSU maturation intermediates containing a large number of different non-ribosomal proteins have been characterized in which the rRNA processing events leading to mature 5.8S and 25S rRNA occur [17], [18]. During maturation many of the non-ribosomal proteins leave the particles [19]. The pre-60S particles move from the nucleolus through the nucleoplasm to the nuclear pore, through which they are released in dependency of various export factors to the cytoplasm [20], [21], [22], [23], [24], [25].

Most of the ribosomal biogenesis factors are essential for growth. Among them are the 4 Noc-proteins and Nop14p, each of which is highly conserved in eukaryotic organisms. Heterodimeric Noc-complexes represent a theme with variations involved in both branches of eukaryotic ribosomal subunit maturation. The Noc1p-Noc2p-complex is associated with early pre-60S ribosomes in the nucleolus whereas Noc2p-Noc3p-complex assembles with later emerging pre-60S particles [26]. Both Noc1p and Noc3p contain a highly conserved stretch of about 45 amino acids with an homologues counterpart found in Noc4p [26], [27]. In contrast to the other Noc-proteins, Noc4p is involved in SSU biogenesis [28]. It can be isolated from yeast extracts in a salt stable SSU-processome submodule together with Nop14p, most likely due to direct protein-protein interactions. Nop14p is another SSU biogenesis factor [28], [29] and component of the pre-90S particle [6]. Depletion of any of the two proteins leads to accumulation of the early-occurring 35S-, and 23S pre-rRNA, whereas the 20S and 27SA_2_ pre-rRNA levels are reduced [28], [29]. Furthermore, Noc4p co-precipitates early pre-18S rRNA [30]. However it is not clear, how the Noc4p-Nop14p subcomplex contributes to the functional architecture of the 90S pre-ribosome.

Here we analyse *in cis*-requirements for incorporation of Noc4p into pre-ribosomes. We analysed a number of deletion and point mutants of NOC4 *in vivo* and identified thereby distinct domains of Noc4p which are required and sufficient for cellular growth, the association with Nop14p and pre-ribosomes or for the efficient cleavage at early rRNA processing sites. The Noc-domain containing C-terminus of Noc4p mediates protein-protein interactions, since a Nop14p-Noc4p complex lacking the N-terminal part of Noc4p can be formed in an heterologous co-expression system. We found that formation of the Noc4p-Nop14p subcomplex and association with pre-ribosomes were always coupled in all the mutants analysed but that on the other hand Nop14p is not strictly required for the incorporation of Noc4p into pre-ribosomes. Replacement of the Noc4p-Noc-domain by its homologues Noc1p-counterpart resulted in a hybrid Noc4p variant which failed to copurify with Nop14p and pre-ribosomes. Remarkably, exchange of 6 aminoacids within the Noc1-Noc-domain of this hybrid Noc4p protein was sufficient to restore its essential *in vivo* functions. These data suggest that Noc-domains of Noc1p and Noc4p share a common structural backbone in which diverging amino acids play crucial roles in formation of regulated interactions, an essential characteristic of Noc-domain containing proteins.

## Results

### Construction and Growth Phenotypes of *Noc4*-Deletion Mutants

**Figure 2:**
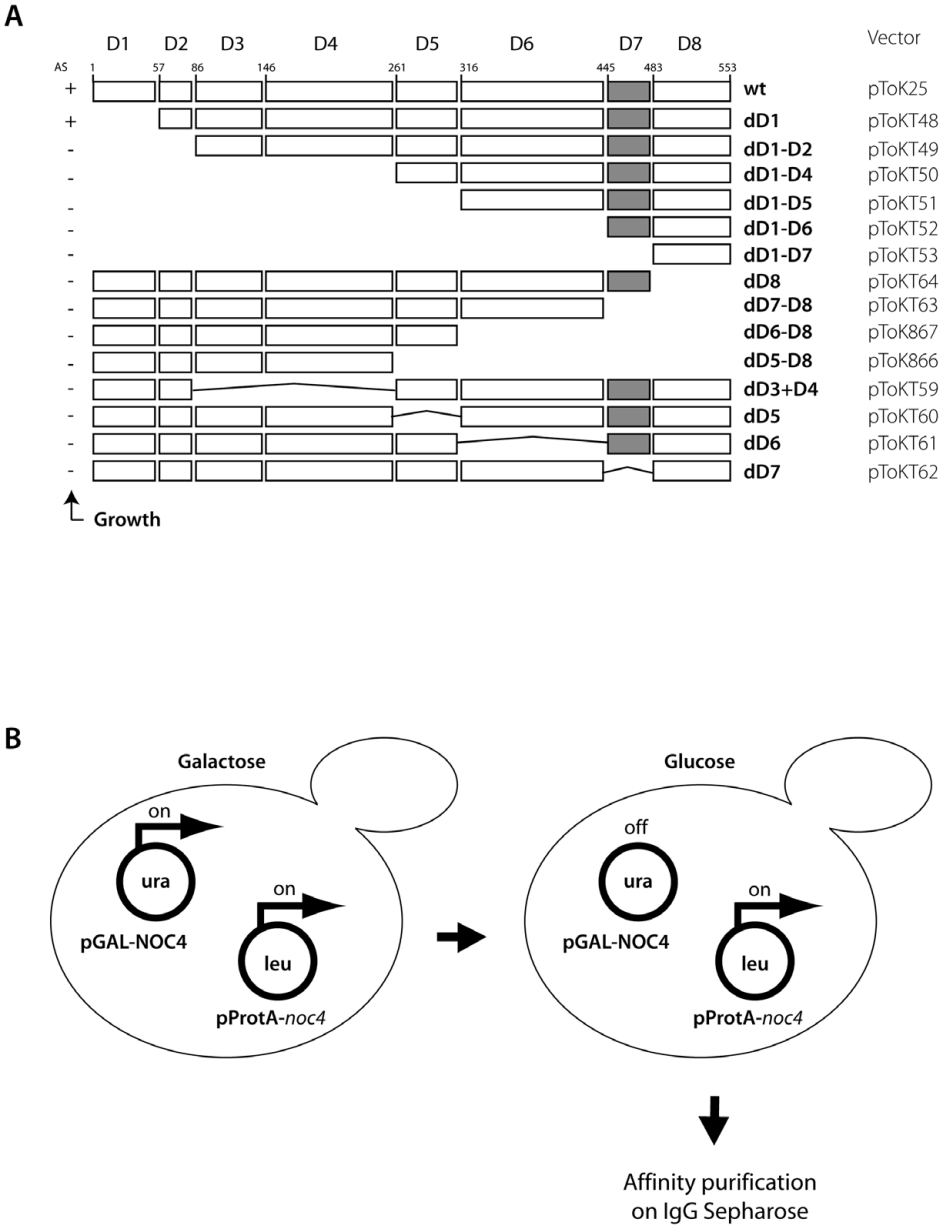
Deletion constructs of NOC4 and expression strategy. (A) NOC4 was divided into eight domains according to their level of conservation with NOC4-homologues of other species. Deletion constructs were cloned in a vector under the control of the constitutive NOP1 promoter and were N-terminally fused to the ProteinA epitope tag. The gray box represents the Noc-domain (D7). (B) Strategy to perform *in vivo* interaction studies with non-viable deletion mutants of NOC4. Yeast strain *Toy489* expressing wildtype NOC4 under the control of the glucose-repressible GAL1/10 promoter was transformed with plasmids coding for truncated Noc4p versions N-terminally fused with a Protein A tag under the control of the constitutive pNop1 promoter. Cells were cultivated in medium containing galactose as carbon source and then shifted for 16 h on glucose containing medium to shut off expression of wildtype NOC4 and to allow truncated Noc4p versions expressed to interact with pre-ribosomal components. ProtA-tagged Noc4p-variants were affinity purified using IgG-sepharose beads. (Note: all strains contained chromosomally tagged Nop14-HA).

Yeast Noc4p is a 63 kDa protein with putative homologues in many eukaryotic, but not archaeal species. It is a protein essential for growth, required for SSU maturation and a component of the Noc4p-Nop14p SSU processome submodule which can be isolated from yeast cells under stringent salt conditions [28]. The goal of this study was to find out whether some of these different functional properties can be related to different domains of Noc4p. Since no tertiary structure information about Noc4p-homologues or related proteins is available we subdivided the Noc4p-primary structure into several subdomains by other means: the different grade of conservation within distinct parts of Noc4p coding sequences from several eukaryotic species served to classify the polypeptide into eight domains (Fig. 2A)(see also Fig. 1 [28]). We generated plasmids supporting the expression of variants of Noc4p with deletions in either one of the eight domains or in combined domains in yeast cells according to the scheme depicted in Fig. 2A. The plasmids were transformed into yeast strain *ToY30* (*Noc4-Shuffle* [28]) which has a full deletion of the coding region of the chromosomal NOC4, but which is complemented by a plasmid carrying wild type NOC4 and the URA3 gene. The ability of the individual constructs to complement the essential NOC4 function was tested by counterselection against the NOC4/URA3 plasmid on 5-FOA-plates. Only one of the 8 domains, coding for the N-terminal 57 aminoacids of Noc4p, was dispensable for growth (Fig. 2A).

### Approach to Study *In Vivo* Association of Truncated Noc4p Variants with Pre-Ribosomal Components

**Figure 3:**
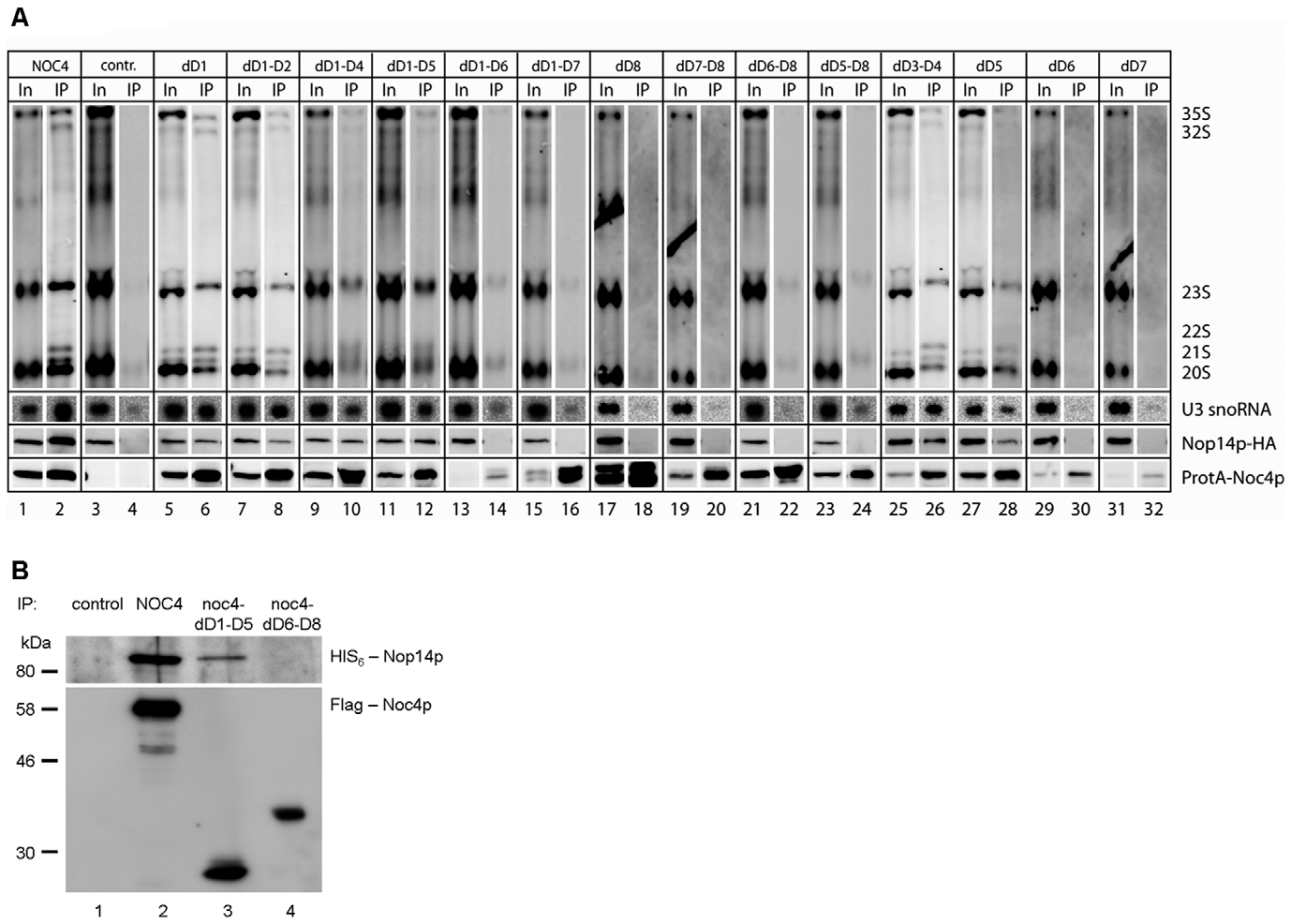
Analysis of pre-ribosomal components associated with Noc4p-variants. (A) Co-immunoprecipitation experiments of wild type cells and mutants expressing truncated Noc4p versions. Logarithmically growing yeast cells which express truncated versions of ProtA-Noc4p under the control of a constitutive promoter and *wildtype* Noc4p under the glucose repressible GAL1/10 promoter were shifted for 16 hours from galactose to glucose to shut off the expression of *wildtype* Noc4p. ProtA-Noc4p variants were affinitypurified and analyzed for association with Nop14p-HA by Western blotting and pre-18S rRNA and U3 snoRNA by Northern blotting. Noc4p-constructs were identified using a ProteinA antibody. Nop14p was detected by an antibody directed against its C-terminal HA epitope. Pre-rRNA and U3 snoRNA-species were detected using probes complementary to the ITS1 (D-A2) region of pre-rRNA's and U3-snoRNA, respectively. Signals in input lanes (In) correspond to 1% of cell extracts loaded onto IgG sepharose beads (IP lanes). (B) The C-terminal domain of Noc4p binds Nop14p when co-expressed in a heterologous baculovirus expression system. Equal numbers of SF21 insect cells were infected with recombinant MultiBac baculoviruses, carrying a HIS_6_-NOP14 allele alone (control) or in combination with different Noc4 alleles (Flag-NOC4, *Flag-noc4-dD1-D5*, *Flag-noc4-dD6-D8*) and incubated for 48 hours. Flag-Noc4p variants were immunoprecipitated from the cell extracts using anti-flag M2 agarose beads and eluted with Flag-peptide. Aliquots of the eluates were analyzed for interactions between the Flag-Noc4p variants and HIS_6_-Nop14p by Western blotting. (Co-)eluted proteins were detected using anti-Flag antibodies or antibodies directed against the HIS-tag, respectively. Equal volumes of eluates were loaded.

To assign which parts of Noc4p are involved in its association with pre-ribosomes, Nop14p and the SSU processome, the following strategy was applied (Fig. 2B): Noc4p-variants N-terminally fused with a ProteinA tag (ProtA-Noc4p) were expressed in yeast strain *ToY489* where wildtype NOC4 is under control of the conditional GAL1/10 promoter and Nop14p is expressed as a C-terminal HA-tag fusion protein (Nop14p-HA). After partial depletion of wildtype Noc4p through 16 hours growth on glucose containing medium (Fig. S1A), Noc4p variants were affinity purified via their N-terminal ProteinA-tag and analysed for their association with precursor rRNA, U3 sno-RNA and Nop14p-HA. As expected, full length ProtA-Noc4p specifically co-precipitated 35S, 32S and 23S pre-rRNAs as well as the U3 snoRNA and Nop14p-Ha (Fig. 3A, compare lanes 1 and 2). Furthermore, a strong association with putative alternative intermediates of the 18S rRNA-maturation pathway (21S- and 22S-pre-rRNA) [31], [32] for discussion} became evident (Fig. 3A, lanes 1 and 2). These data confirm that Noc4p is in vivo associated with early pre-ribosomal particles [6] including 21S rRNA and 22S rRNA containing ribosomal particles [30], the SSU-processome [11] and its most likely direct proteinaceous interaction partner Nop14p [28].

### *In Vivo* Association of N-Terminally Truncated Noc4p-Variants with Pre-Ribosomal Components

Next, we analysed how N-terminal deletions (domains 1–5) of NOC4 affects the association of Noc4p with pre-ribosomal components. The *in vivo* association of the resulting Noc4p variants with U3 snoRNA and Nop14 and SSU pre-rRNAs (Fig. 3A, lanes 6, 8, 10, 12, 26, 28) was slightly weakened, but a significant amount of all these components could still be co-precipitated. No pronounced difference in pre-rRNA association is visible between Noc4p variants in which either Domain 1, Domain 1 and 2, Domain 3 and 4, Domain 5, Domain 1 to 4 or Domain 1 to 5 are lacking, although in contrast to the deletion of Domain 1 the latter truncations are all lethal. Therefore, the slightly reduced level of pre-rRNA association of Noc4p variants in these mutants is probably not responsible for their failure to support growth. Instead, it is likely that the lack of Domain 2 to 5 affects other essential functions of Noc4p.

All 5 N-terminally truncated variants of Noc4p as well as full length Noc4p, coprecitated 21S and 22S rRNA (Fig. 3A, lanes 2, 6, 8, 10, 12, 26, 28) suggesting that they are associated with 21S and 22S rRNA containing pre-ribosomes.

### The C-Terminal Domain of Noc4p Represents a Protein-Protein Interaction Module and Is Required and Sufficient for Its Association with Nop14p, U3 snoRNA and Pre-Ribosomes

The previous experiments indicated that the 5 N-terminal domains of Noc4p are not strictly required for its incorporation into pre-ribosomal particles. The Noc4p variant consisting of the 237 C-terminal amino acids of Noc4p and thereby lacking these 5 domains, appeared to be capable of coprecipitating Nop14, pre-18S rRNA and U3 snoRNA. On the other hand, truncation of any of the C-terminal domains 6 to 8 resulted in an apparent loss of association with Nop14p-HA, 18S rRNA precursors and U3 snoRNA (Fig. 3A, lanes 14, 16, 18, 20, 22, 24, 30 and 32). When we immunoprecipitated HA-tagged Nop14p with anti HA antibodies from the corresponding cellular extracts, corroborative results were obtained: HA tagged Nop14p could only coprecipitate Noc4p variants which did not lack its three C-terminal domains 6 to 8 (Fig. S1B). To prove that the C-terminal part of Noc4p represents a protein-protein interaction domain required for Nop14p binding we co-expressed either full length or the N-terminal or C-terminal truncation together with full length Nop14p in a baculovirus expression system. While both Flag-tagged full length Noc4p and the C-terminal part of Noc4p co-precipitated significant amounts of Nop14p, the N-terminal Noc4p part failed to form a complex with Nop14p (Fig. 3B).

These data indicated that the C-terminal 237 amino acids of Noc4p are sufficient to bind Nop14p and required for incorporation of Noc4p into pre-ribosomal particles. We conclude that the N- and the C-terminal domain of Noc4p exhibit different functions. The C-terminal part of Noc4p represents an important interface for the incorporation of Noc4p into early pre-ribosomes and the SSU-processome and for formation of the stable Noc4p-Nop14p SSU-processome submodule. When recruited to 40S pre-ribosomal particles via the C-terminus, the N-terminal part seems then to facilitate the U3-dependent processing of the 18S rRNA precursor.

### Role of Nop14p for Incorporation of Noc4p into Pre-Ribosomes

**Figure 4:**
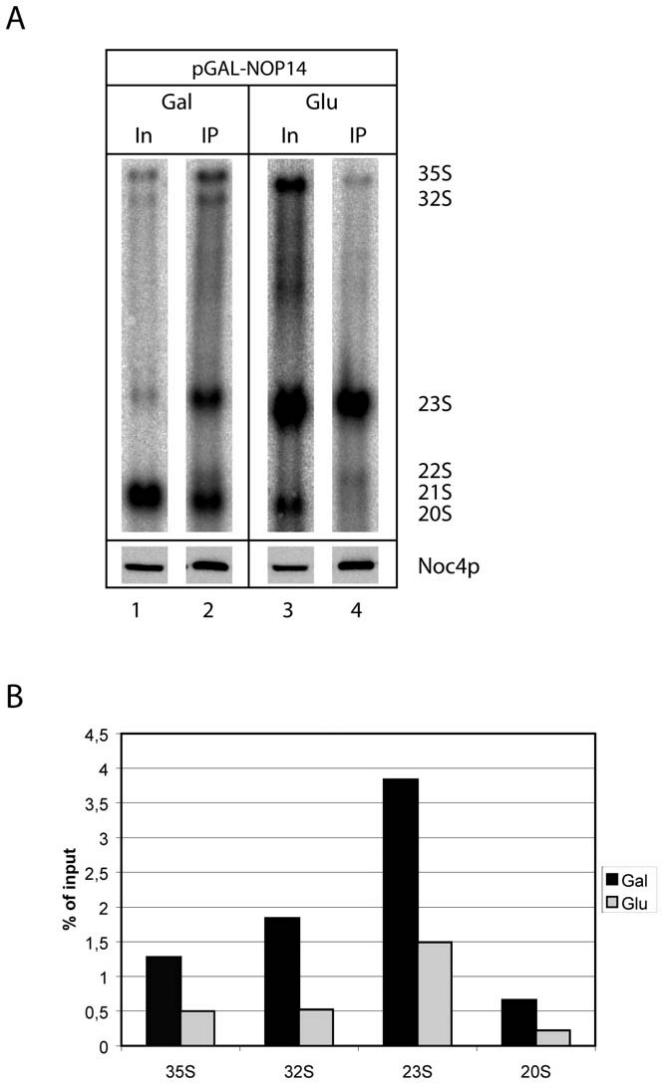
Depletion of Nop14p results in reduced Noc4p interaction with pre-ribosomes. (A) Logarithmically growing yeast cells expressing NOP14 from a vector under the control of the glucose repressible GAL1/10 promoter were inoculated in YP-glucose containing medium for 16 h. Cell extracts before (lane 1) and after (lane 2) shift to glucose were prepared and equal amounts of protein were loaded onto IgG sepharose beads. Aliquots of the affinity purified fractions were analyzed by Western blotting for presence of Nop14p and by Northern blotting to determine association with pre-ribosomal RNAs (lanes 2 and 4). rRNA species were determined using probes complementary to the ITS1 (D-A2) and Noc4p was detected using a monoclonal Noc4p-antibody. (B) Quantitation of the Northern blot depicted in (A). The levels for each rRNA-species are normalized to the input. Signals in input lanes (In) correspond to 1% of cell extracts loaded onto IgG sepharose beads (IP lanes).

Interestingly, for none of the Noc4p-variants analysed the association of Noc4p with pre-ribosomes and with Nop14p was uncoupled. Possible explanations for this finding are that Noc4p-incorporation into the SSU-processome either depends on or is a prerequisite for its (most likely) direct interaction with Nop14p, or that Noc4p domains which mediate the interaction with Nop14p play additional independent roles in recruitment of Noc4p into pre-ribosomes. To exclude one or the other of these alternatives, we analysed whether Noc4p can associate with pre-ribosomal subunits in the absence of Nop14p. Therefore, we generated strain *ToY559* in which NOP14 is expressed under the control of the GAL1/10 promoter and Noc4p is encoded in fusion with a C-terminal ProtA tag (Noc4p-ProtA). After 16 hours shift to glucose-containing medium, we observed accumulation of 35S and 23S pre-rRNAs whereas 20S pre-rRNAs were significantly reduced (compare lanes 1 and 3, Fig. 4A) confirming that Nop14p is involved in an early step of pre-40S maturation. Noc4p-ProtA was affinity purified from cellular extracts and associated 18S rRNA precursors were analysed by Northern blotting (Fig. 4A). Although some 18S rRNA precursors copurified less efficiently with Noc4p-ProtA after previous *in vivo* depletion of Nop14p (Fig. 4A, compare input lanes 1 and 3 with IP-lanes 2 and 4) the total amount of Noc4p-copurifying 18S pre-rRNAs was not strongly reduced. In particular, the absolute amount of 23S and 35S pre-rRNA, RNA's which strongly copurify with Noc4p in wildtype conditions, but not the Noc4p-level, are largely increased in extracts from cells depleted of Nop14p (Fig. 4A, compare input lanes 1 and 3, [29], arguing that the somewhat reduced efficiency of pre-rRNA copurification in this situation could be due to their consequent relative excess over Noc4p (Fig. 4B). Altogether, this suggests that formation of a Noc4p-Nop14p subcomplex is not strictly required for Noc4p's incorporation into pre-ribosomes.

### Conserved Amino Acids in the “Noc-Domain” of Noc4p Are Crucial for Its Incorporation into Pre-Ribosomes

**Figure 5:**
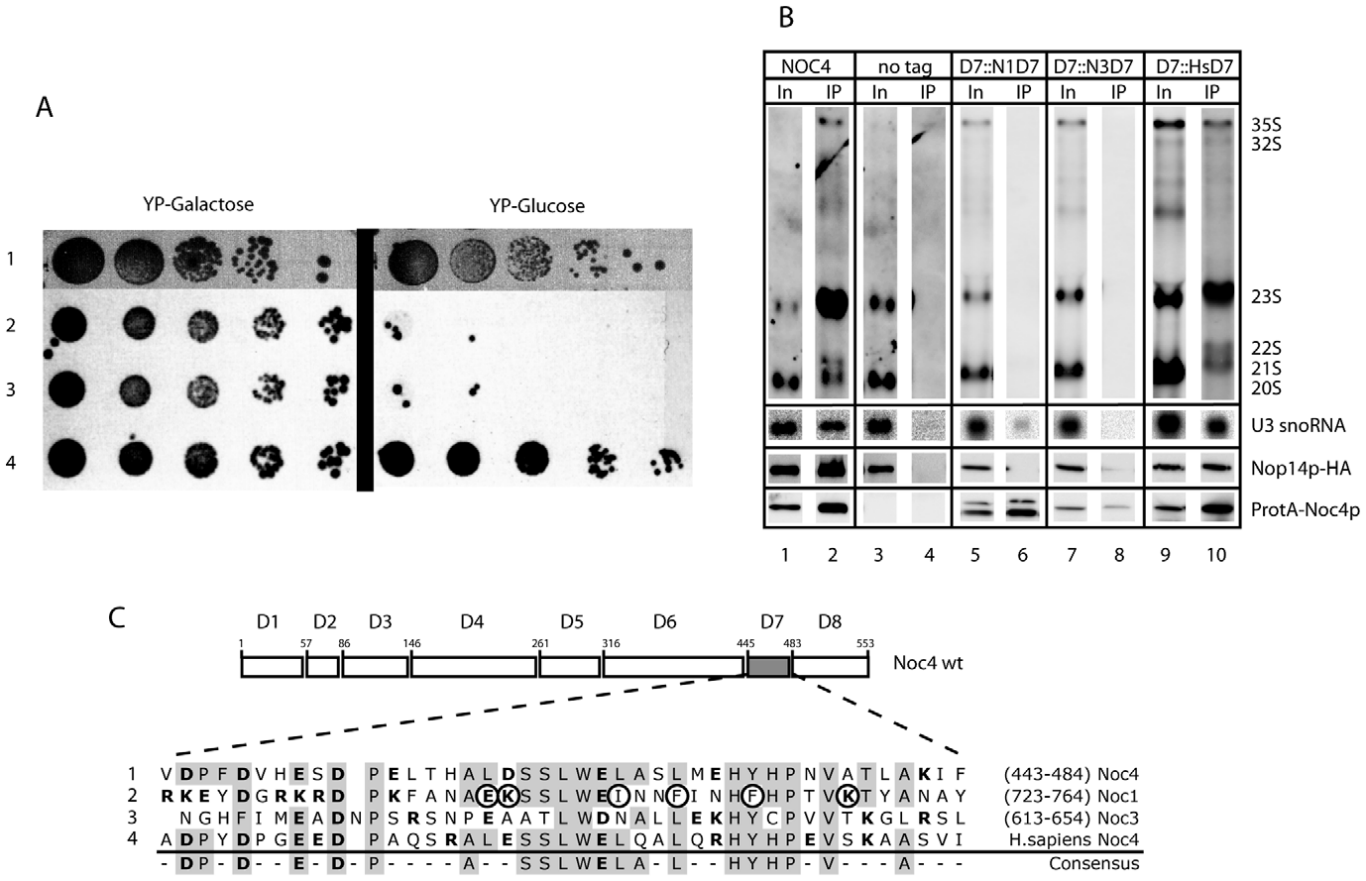
Exchange of Noc-domains within yeast Noc4p and determination of pathway-specific amino acids. (A) The Noc-motif from Noc4p was substituted with the corresponding stretch of Noc1p, Noc3p or the putative Noc4p-homologue of *H. sapiens*, respectively. The hybrid alleles were cloned into a vector supporting their constitutive expression in yeast and the resulting plasmids (pTOKT87, pTOKT88, pTOKT89) were transformed into strain *Toy489* expressing wildtype Noc4p under the control of the GAL1/10 promoter. Serial dilutions of yeast cells grown for 3 days on plates containing galactose or glucose as carbon source are shown. 1: NOC4 wildtype. 2: Noc4p-Noc-domain replaced by Noc1-Noc-domain. 3: Noc4p-Noc-domain replaced by Noc3-Noc-domain. 4: Noc4p-Noc-domain replaced by the corresponding stretch of human Noc4p. (B) Cells expressing Noc4p under the control of the pGAL1/10 promoter (*Toy489*) and Noc4p-hybrids N-terminally decorated by an ProteinA-tag under the constitutive pNop1 promoter were shifted for 16 h from galactose containing medium to glucose containing medium to shut down expression of wild type NOC4. Cell extracts were prepared and equal amounts of protein were loaded onto IgG sepharose beads. Aliquots of the precipitates were analyzed for association of Protein tagged Noc4p variants with Nop14p (Western blot) and to determine copurification with pre-ribosomal RNAs and U3 snoRNA (Northern blot). Nop14p was detected by an antibody directed against its C-terminally fused HA epitope, Noc4p-hybrid constructs via its N-terminal fused ProteinA tag. Pre-rRNA species were determined using probes complementary to the ITS1 (D-A2) region of pre-rRNA, U3 snoRNA was detected with a complementary oligo probe. Signals in input lanes (In) correspond to 1% of cell extracts loaded onto IgG sepharose beads (IP lanes). (C) Sequence comparison of Noc-domains analysed in this study. Lanes 1–3 shows the sequences of the Noc-domains of Noc1p, Noc3p and Noc4p from *S. cerevisiae*, respectively, lane 4 shows the corresponding sequence region of the putative human Noc4p homologue. Exchange of the six encircled residues in the Noc1p sequence with the corresponding aminoacids in Noc4p sequence resulted in a hybrid Noc4p - Noc1p allele which is able to complement the essential functions of of yeast NOC4.

The C-terminus of Noc4p contains a stretch of about 45 amino acids (Domain 7 =  “Noc-domain”) which is highly conserved in predicted Noc4p homologues of other eukaryotic species. Remarkably, two other yeast proteins, Noc1p and Noc3p, which are required for LSU maturation [26], contain each a region homologous to the Noc4p-Noc-domain [27], [28]. The respective conservation on primary structure level (Fig. 5C), and secondary structure predictions [27] would suggest that these different Noc-domains fold in a common structural backbone. Deletion of the Noc-domain coding region (Domain 7, Fig. 2) is lethal for yeast cells and interferes with incorporation of Noc4p into SSU precursors (Fig. 3A). We wondered, whether homologous coding regions of the yeast Noc4p- Noc-domain found in either yeast Noc1p and Noc3p or in predicted counterparts of Noc4p in other eukaryotic species could overtake its essential function. Therefore we constructed plasmids which support constitutive expression of ProteinA-tagged yeast Noc4p variants with its Noc-domain replaced by the counterparts from yeast Noc1p (pToKT87), yeast Noc3p (pToKT89) or the predicted human Noc4p (pToKT88) (Fig. 5C). The vectors were then transformed into yeast strain *ToY489* (*pGAL-NOC4*) where cellular expression of Noc4p is under the control of the GAL1/10 promoter. When transferred to glucose containing plates, cells expressing Noc4p-Noc1p or Noc4p-Noc3p hybrids stopped growth, while the plasmids coding for yeast Noc4p or for the hybrid of yeast Noc4p and human Noc4p supported cellular growth (Fig. 5A). In addition, immuno-precipitation experiments showed that the hybrid of human and yeast Noc4p but not the Noc4p-Noc1p and Noc4p-Noc3p hybrids associated with Nop14p and were incorporated into SSU precursor particles (Fig. 5B). These results suggest that the essential role of yeast Noc4p-Noc-domain in the Noc4p-Nop14 subcomplex formation and SSU-precursor incorporation is conserved in eukaryotic evolution. On the other hand, diverging amino acids in Noc-domains found in yeast Noc4p and Noc1p and Noc3p seem to mediate functions specific to these individual proteins.

We compared which aminoacids in the Noc-domain of yeast and human Noc4p are conserved or similar but differ from the yeast Noc1p-Noc-domain (see Fig. 5C). Replacement of these six aminoacids of the Noc1p-Noc-domain by the ones from Noc4p-Noc-domain was sufficient to restore the ability of the resulting Noc4p-Noc1p hybrid to complement for the essential functions of NOC4 (Fig. S2).

This further strengthens the assumption that Noc-domains found in yeast Noc-proteins share a common structural backbone in which individual amino acids are important for specific functions of Noc proteins, as the formation of protein complexes with Nop14p or Noc2p and the regulated interaction with pre-ribosomal particles.

## Discussion

The experiments shown confirm that Noc4p is part of the SSU-processome, since it 1) copurifies with U3 sno-RNA and 2) is required for SSU maturation (Fig. 3A, [6], [7], [8], [11]). Nevertheless, it was not identified to be part of one of the major SSU-processome subcomplexes, namely the U3 snoRNA core particle [33], the Mpp10p complex, the UTP-A/t-UTP complex, the Pwp2/UTP-B complex and the UTP-C complex [7], [8]. Each of these subcomplexes can be either isolated from yeast cell extracts as separate entity [7] and/or some of their human counterparts (hImp3p, hImp4p, hMpp10) can be reconstituted in vitro from individual components [34]. The main protein copurifying with tagged Noc4p in *ex vivo* affinity purification experiments under stringent high salt conditions was the SSU-processome component Nop14p [28] suggesting that the Noc4p/Nop14p heterodimer represents another architectural submodule of the SSU processome.

*In vivo* depletion of Nop14p does not completely block association of Noc4p with pre-ribosomal particles (Fig. 4) indicating that direct interaction of Noc4p with Nop14p is most likely not strictly required for its incorporation into the SSU-processome. On the other hand, for none of the different Noc4p mutants analysed the interaction with Nop14p and the association with pre-ribosomes were uncoupled. The experiments indicate that the C-terminal part of Noc4p is sufficient and that the integrity of the Noc-domain contained therein (Fig. 4 and Fig. 5) is required for association of Noc4p with both Nop14p and SSU precursors. Accordingly, the C-terminus of Noc4p is important for two kind of interactions, a salt stable one with Nop14p and a salt labile interaction with residual SSU-processome components, each of which can persist independent on the other ([28], Fig. 4). Our results provide evidence that association of Noc4p with pre-ribosomes can occur without the presence of Nop14p (Fig. 4), but future studies will have to show whether formation of the salt stable Noc4p-Nop14p SSU-processome subcomplex requires the presence of other SSU-processome components. Aminoacids which are conserved in Noc-domains of Noc4p from yeast and human but are diverged in the Noc-domains of Noc1 and Noc3p seem to be important for the establishment of these different kind of interactions. Based on structure prediction algorithms it was suggested that Noc-domains and large parts of Noc-proteins fold in alpha helical repeats similar as seen in HEAT (Huntington-elongation-A_-subunit-TOR) repeat proteins [27]. HEAT repeats found in the importin-β tpye of nucleo-cytoplasmic transport proteins are thought to allow regulated formation of timely transient protein-protein interaction interfaces [35]. Our data now show that the Noc-domain containing C-terminus of Noc4p represents an interface which is sufficient to bind Nop14p. It was estimated that more than 0.2% of eukaryotic proteins contain HEAT-like repeats which indicates that this abundancy reflects a functional versatility perhaps in establishing various protein-protein interactions [36]. Future studies will have to confirm the existence of HEAT repeat-like folds in Noc4p and to explore their role in mediating the direct protein-protein interactions with Nop14p or possibly other proteinaceous factors and in the regulated association of Noc4p with pre-18S rRNA containing RNPs.

Interestingly, N-terminal truncated Noc4p variants, like full length Noc4p (Fig. 3A), associate with 22S- and 21S pre-rRNA. Apparently, in growing wildtype yeast cells 21S pre-rRNA and 22S pre-rRNA are very short-lived but nevertheless, they are associated in significant amounts not only with Noc4p but also with other SSU-processome components, e.g. Utp10 and Utp20 [13] under these conditions. The short half life of 21S and 22S rRNA could be either explained by their rapid degradation or by a fast and productive maturation into 18S rRNA. Accordingly, Dez et al. suggested that the very stable association of Utp10 and Utp20 with 21S and 22S and 23S pre-rRNA containing RNP's might be part of a mechanism that targets these particles to Tramp5 and exosome dependent degradation [13]. On the other hand, Vos et al. showed that in certain yeast mutants with no apparent growth defect, production of functional small ribosomal subunits completely depends on a rRNA processing pathway which involves 21S rRNA as an intermediate [31].

## Materials and Methods

### Yeast Cell Culture, Strain Construction and Plasmid Construction

For cultivation transformation, mating, sporulation, preparation of genomic DNA, and tetrade dissection of yeast, standard protocols were followed [37]. 5-FOA resistant clones and plasmide shuffling experiments were carried out on YNB supplemented with glucose or galactose, respectively and the amino acids required, in the presence of 1 g/l of 5-FOA (Toronto Research). Construction of yeast strain *Toy30/Noc4 Shuffle* was described before [28]. To construct strain *Toy489* (his3­1,leu2-0,ura3­0,ypr144c::KANMX4, YDL148c-HA-HIS3MX6, pToK401) in strain *ProtA-NOC4/ToY31* [28] a 3xHA tag coding sequence was integrated at the NOP14 gene locus by homologous recombination according to [38]. (Oligo sequence information is available upon request). The resulting strain was transformed with plasmid pToK401 (Ycplac33/pGAL-NOC4) and after several days of growth on medium lacking uracil it was screened for leucine auxotroph clones. To create *ToY559* (his3­1, leu2-0, ura3­0, YPR144C-TAP-URA3, ydl148c::kanMX4, pToK495), Euroscarf strain Y23846 (his3­1/his3­1, leu2­0/leu2­0, lys2­0/LYS2, met15­0/MET15, ura3­0/ura3­0, YDL148c::kanMX4/YDL148c) was transformed with plasmid pToK3 (Ycplac33/NOP14) and the resulting genetic offsprings were analysed by tetrade analysis. A geneticine resistant and 5-FOA sensitive clone was then transformed with plasmid pToK495 (Ycplac111/pGAL-NOP14), it was selected for resulting clones resistant to 5-FOA on galactose containing medium and a TAP-tag coding sequence was integrated at the NOC4 gene locus by homologous recombination according to [39]. (Oligo sequence information is available upon request). For details on construction of plasmids used in this study see supplementary tables Table S1 and Table S2.

### Affinity Purification Experiments

200 ml of cells from of a logarithmically growing culture of a yeast strain containing a ProteinA tagged version of Noc4p were harvested and the cell pellet was washed with 10 ml ice cold H_2_O. Cells were disrupted by bead beating in ice cold buffer A200 (200 mM KCl, 20 mM Tris pH 8.0, 5 mM MgAc, 1 mM DTT, 1 U/µl RNAsin (RNAse inhibitor), 1 mM PMSF, 2 mM Benzamidin) on a vibrax for 45 min at 4°C. The lysate was then centrifuged at 15000 g for 10 min and the supernatant was transferred into a new cup. The protein concentration was determined by Bradford. 30 µl of IgG-Sepharose (GE) slurry were washed in batch with 15 ml H_2_O, followed by two times washing with 15 ml buffer A200. 10 mg of whole cell extract was loaded on the beads and incubated for >1 h at 4°C on a turning wheel. Beads were then transferred into a micro column (BioRad) and washed 1×1 ml, 5×2 ml and 1×10 ml with buffer A200. 20% of the beads were used for protein analysis, the rest was used for RNA extraction. Each 200 µg of the whole cell extract were withdrawn for RNA-analysis. Protein A-tagged Noc4p variants and HA-tagged Nop14 were immunodetected after western blotting by an peroxidase anti-peroxidase immunocomplex (Dako Cytomation) (dilution 1∶5000) and monoclonal rat anti HA antibody 3 F10 (Roche Diagnostics) (dilution 1∶10.000).

### Baculoviral Protein Expression and Purification

Recombinant expression of Noc4p and Nop14p was performed in *Spodoptera frugiperda* insect cells using the Multibac system described previously [40], [41], [42]. NOC4 and deletion mutant alleles *noc4-dDx* carrying an N-terminal Flag-tag were cloned in the acceptor plasmid pFL, whereas NOP14 carrying an N-terminal 6xHis-tag was cloned in the donor plasmid pSPL. The respective PCR products were cloned in the target vectors using restriction enzymes SalI and PstI.

The donor plasmid pSPL-6xHis-NOP14 was fused to the different pFL-Flag-NOC4*(-dDx*) acceptor plasmids via *in vitro* Cre-loxP reaction using Cre-recombinase (New England Biolabs). For protein expression, 25×10^6^ insect cells were infected for 48 h, harvested at 136 g for 10 min and lysed in 25 ml lysis buffer (50 mM Tris/HCl, pH 7.5; 100 mM NaCl, 5 mM MgCl_2_, 0.15% NP40, 1 mM DTT, 1 mM PMSF, 0.5 mM Benzamidine) by three freeze-thaw cycles and subsequent sonification.

Flag-tagged Noc4p protein variants were immunoprecipitated using M2-anti-Flag-Agarose (Sigma-Aldrich) and eluted with 0.2 mg/ml Flag-peptide in lysis buffer. Protein levels of 6xHis-Nop14p and Flag-Noc4p variants in the eluates were analyzed by Western blot using INDIA-His-Probe-HRP (Pierce Biotechnology) and a polyclonal rabbit anti-Flag antibody (Sigma).

### RNA Analysis and Northern Hybridisation

RNAs from total cellular lysates or affinity purified fractions were prepared and analysed as described before [16], pre-18S rRNA was detected on Northern blots by a digoxigenin labeled RNA probe. For this, an ITS1 DNA fragment with 5′ fused T7 promoter sequence was amplified by PCR from yeast genomic DNA with oligos O441 (5′TAA TAC GAC TCA CTA TAG GGT GTA TTG AAA CGG TTT TAA TTG TCC3′) and. O378 (5′GTT TTG GCA AGA GCA TGA GAG C). The amplified DNA served as template to produce a digoxigenine labelled RNA probe according to the manufacturer's protocol of the DIG Northern Starter Kit (Roche Diagnostics). For ITS1 detection, blots were hybridized with this probe in 50% formamide, 5X SSC, 0,5% SDS, 5X Denhards solution at 64°C. Detection of the probe was performed with an anti-Dig antibody coupled to alkaline phosphatase, CDP-star chemi-luminescence substrate (Roche Dignostics) and Fuji LAS Reader 3000. U3 snoRNA was detected with ^32^P 5′ end labelled oligonucleotide O202 (5′ CCA ACT TGT CAG ACT GCC ATT 3′). Hybridisation conditions were 50% formamide, 5X SSC, 0.5% SDS, 5X Denhards solution and 37°C. Radioactive signals were detected by phosphor imaging on the BAS1000 (Fuji).

## Supporting Information

Figure S1 (0.53 MB DOC)

Figure S2 (0.25 MB DOC)

Table S1 (6.45 MB TIF)

Table S2 (5.46 MB TIF)
