# Supplementary material for: The Noc-Domain Containing C-Terminus of Noc4p Mediates Both Formation of the Noc4p-Nop14p Submodule and Its Incorporation into the SSU Processome
Source: PLoS One. 2009 Dec 18;4(12):e8370. doi: 10.1371/journal.pone.0008370 (PMC2794458; doi:10.1371/journal.pone.0008370)
Supplement: Figure S1 — (0.53 MB DOC) [file pone.0008370.s001.doc]

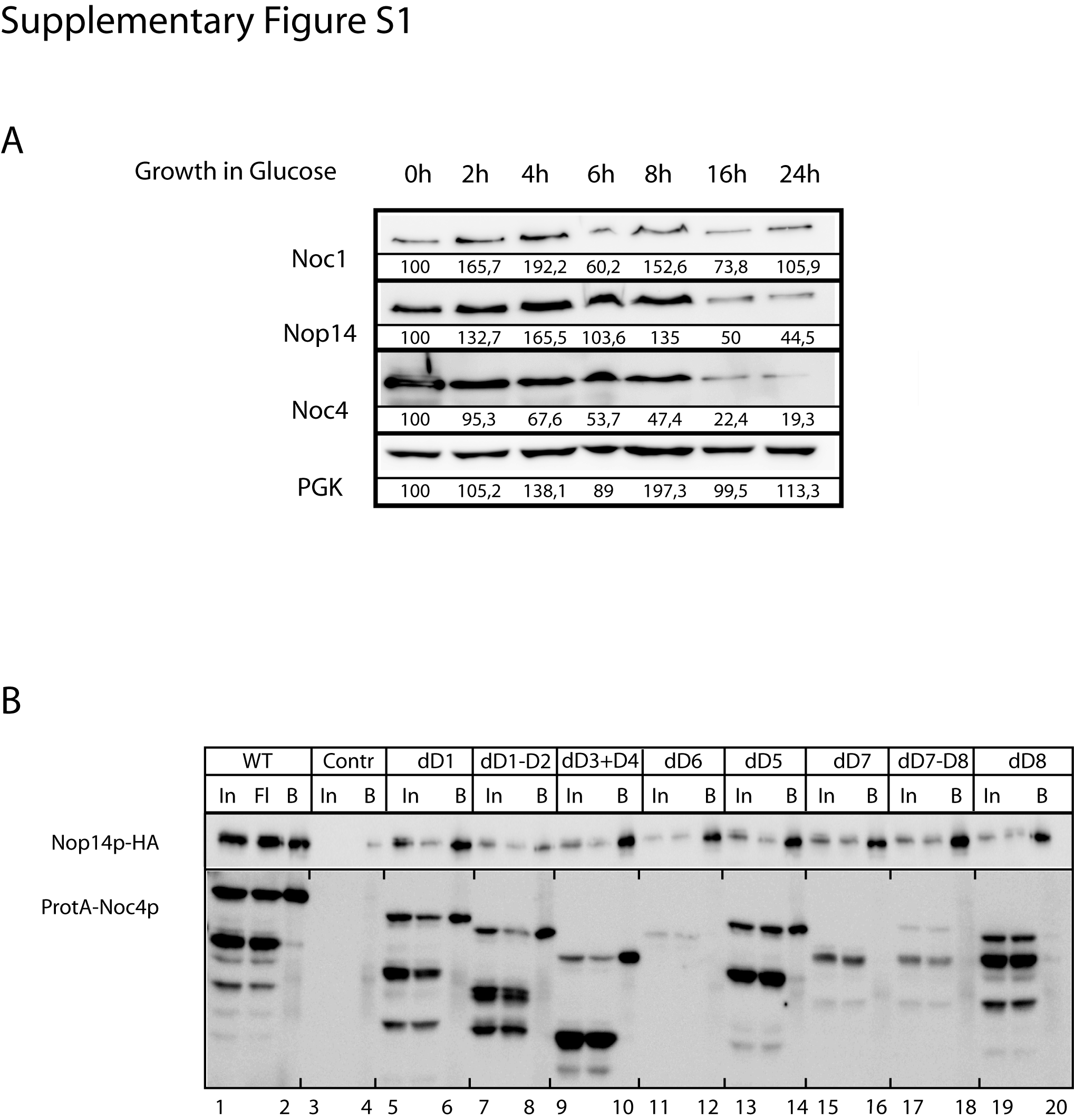


(A) **Depletion kinetics of Noc4p**. At the indicated time points, samples from yeast cultures (*Toy489*) were taken and cell extract was prepared and analysed on a Western blot using polyclonal antibodies against Noc1p (1), monoclonal anti-Noc4p antibodies, anti-HA-antibodies (Nop14p-HA) (clone 3F10, Roche) and monoclonal antibodies (Invitrogen) directed against PGK (3-phospho glycerate kinase) to determine the level of the indicated proteins. Relative levels of signal intensities are indicated.

(B) **Coimmunoprecipitation of Nop14-HA with Noc4p-deletion constructs**. In = input, FL = supernatant of the beads after incubation, B = beads. Same cellular extracts were used as in Fig. 3. Immunoprecipitation was performed using Anti-HA antibodies (3F10, Roche) coupled to ProteinG-Sepharose.

1**. Milkereit, P., O. Gadal, A. Podtelejnikov, S. Trumtel, N. Gas, E. Petfalski, D. Tollervey, M. Mann, E. Hurt, and H. Tschochne**r. 2001. Maturation and intranuclear transport of pre-ribosomes requires Noc-proteins. Cel**l 10**5:499-509.
