# Supplementary material for: The Noc-Domain Containing C-Terminus of Noc4p Mediates Both Formation of the Noc4p-Nop14p Submodule and Its Incorporation into the SSU Processome
Source: PLoS One. 2009 Dec 18;4(12):e8370. doi: 10.1371/journal.pone.0008370 (PMC2794458; doi:10.1371/journal.pone.0008370)
Supplement: Figure S2 — (0.25 MB DOC) [file pone.0008370.s002.doc]

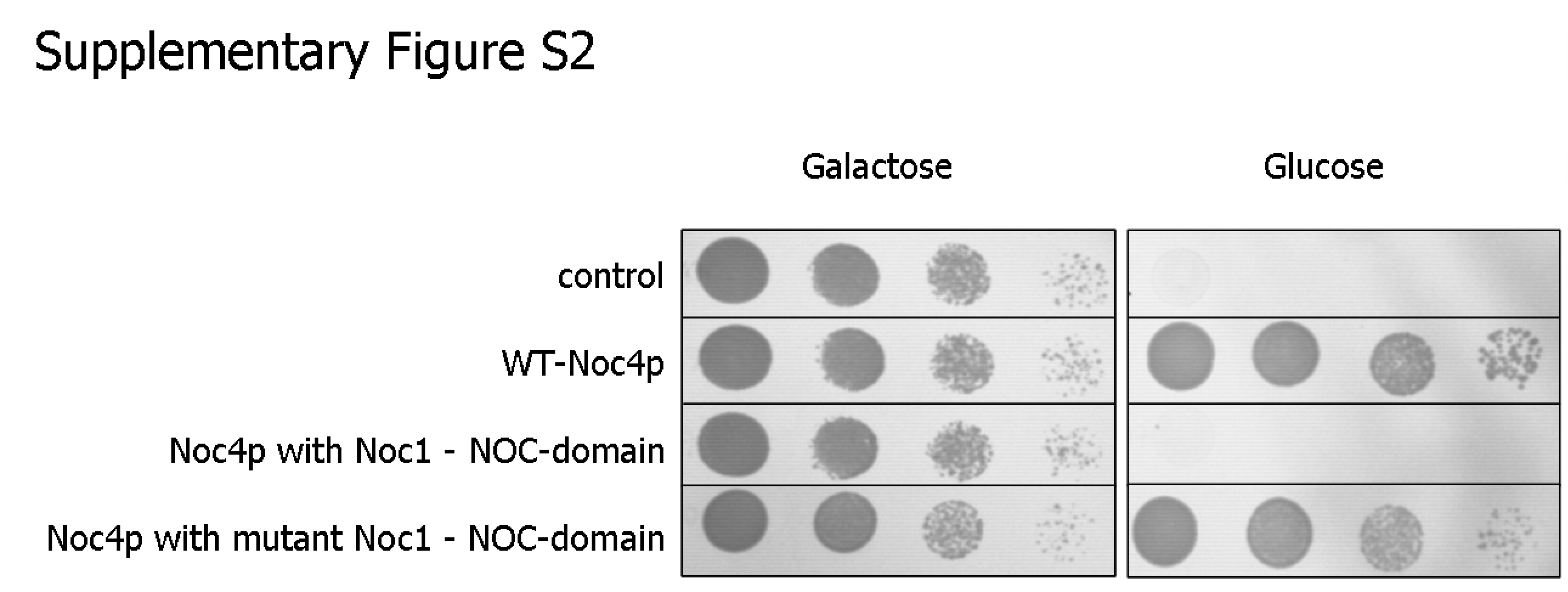


**A mutated Noc1-Noc domain in which 6 aminoacids were exchanged can complement for the Noc4-Noc domain.** The Noc-motif from Noc4p was substituted with the corresponding stretch of Noc1p, or the Noc1-NOC domain in which the following aminoacids were exchanged: E459L, K460D, I466L, F469L, F473Y, A478K (Noc4p with mutant Noc1-NOC-domain). The hybrid alleles were cloned into a vector supporting their constitutive expression in yeast and the resulting plasmids (pTOKT87, pTOKT780) were transformed into strain *Toy489* expressing wildtype Noc4p under the control of the GAL1/10 promoter. Serial dilutions of yeast cells grown for 3 days on plates containing galactose or glucose as carbon source are shown.
