# Supplementary figures and images for: The Noc-Domain Containing C-Terminus of Noc4p Mediates Both Formation of the Noc4p-Nop14p Submodule and Its Incorporation into the SSU Processome
Source: PLoS One. 2009 Dec 18;4(12):e8370. doi: 10.1371/journal.pone.0008370 (PMC2794458; doi:10.1371/journal.pone.0008370)

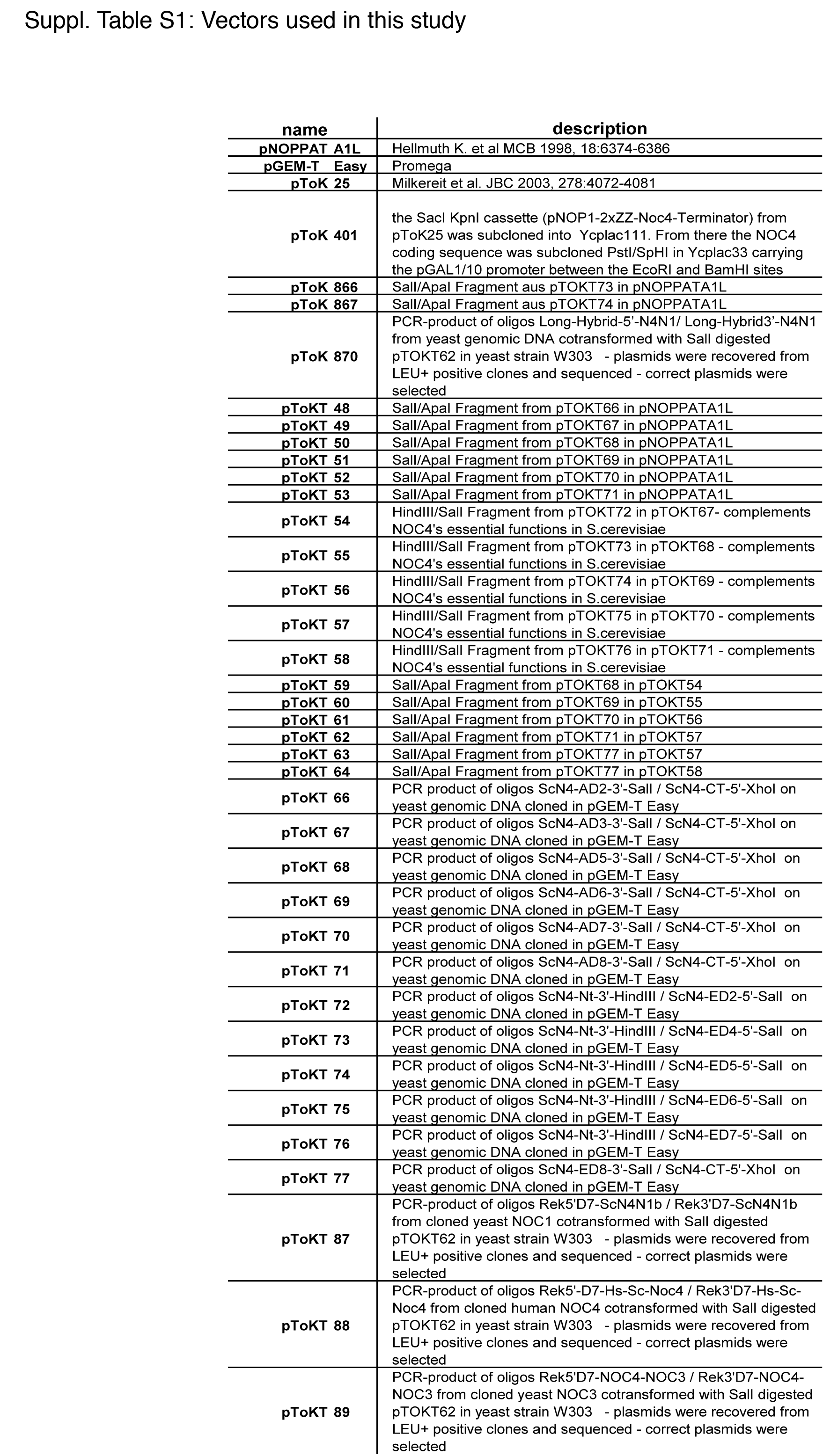

Supplement: Table S1 — (6.45 MB TIF) [file pone.0008370.s003.tif]

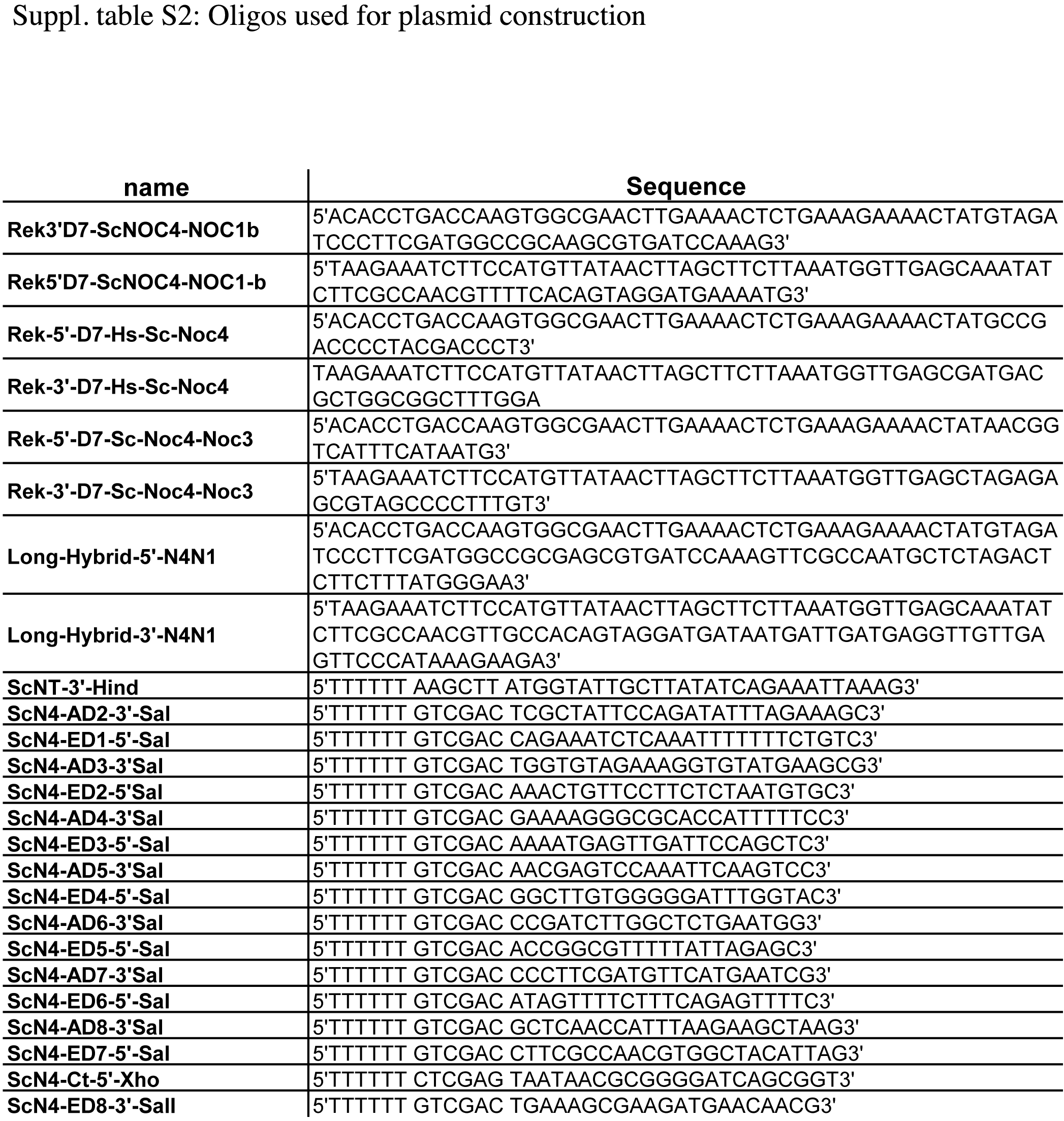

Supplement: Table S2 — (5.46 MB TIF) [file pone.0008370.s004.tif]
